# Attention-deficit/hyperactivity disorder (ADHD) symptoms and their relation to diagnosed ADHD, sociodemographic characteristics, and substance use among patients receiving opioid agonist therapy: a Norwegian cohort study

**DOI:** 10.1186/s12888-023-04980-w

**Published:** 2023-06-29

**Authors:** Jørn Henrik Vold, Anne Halmøy, Fatemeh Chalabianloo, Marianne Cook Pierron, Else-Marie Løberg, Kjell Arne Johansson, Lars Thore Fadnes

**Affiliations:** 1 Department of Addiction Medicine, Haukeland University Hospital, Jonas Lies Vei 65, N-5021 Bergen, Norway; 2 Department of Global Public Health and Primary Care, University of Bergen, Bergen, Norway; 3 Division of Psychiatry, Haukeland University Hospital, Bergen, Norway; 4 Department of Clinical Medicine, University of Bergen, Bergen, Norway; 5 Department of Clinical Psychology, University of Bergen, Bergen, Norway

**Keywords:** Opioid substitution treatment, Attention-deficit/hyperactivity disorder symptoms, Substance-related disorders, Injecting substance use, Adult ADHD self-report scale

## Abstract

**Background:**

Attention-deficit/hyperactivity disorder (ADHD) symptoms may challenge sufficient treatment of substance use and mental disorders. The literature on the extent of such symptoms among patients receiving opioid agonist therapy (OAT) is scarce. This study examined ADHD symptoms using the ADHD self-report scale (ASRS) and the association between the ‘ASRS–memory’ and ‘ASRS–attention’ scores and substance use and sociodemographic characteristics among patients receiving OAT.

**Methods:**

We used data from assessment visits of a cohort of patients in Norway. In total, 701 patients were included from May 2017 to March 2022. All patients responded at least once to two ASRS questions assessing memory and attention, respectively. Ordinal regression analyses were performed to investigate whether the two obtained scores were associated with age, sex, frequent substance use, injecting use, housing status, and educational attainment at baseline, i.e., the first assessment, and over time. The results are presented as odds ratios (OR) with 95% confidence intervals (CI). Additionally, a subsample of 225 patients completed an extended interview, including the ASRS–screener and collection of registered mental disorder diagnoses from the medical records. Standard cutoffs were used to define the presence of each ASRS symptom or a positive ASRS–screener (‘ASRS–positive’).

**Results:**

At baseline, 428 (61%) and 307 (53%) patients scored over the cutoffs on the ‘ASRS–memory’ and ‘ASRS–attention,’ respectively. Frequent cannabis use was associated with higher ‘ASRS–memory’ (OR: 1.7, 95% CI: 1.1–2.6) and ‘ASRS–attention’ (1.7, 1.1–2.5) scores compared with less or no use at baseline, though reduced score on the ‘ASRS–memory’ over time (0.7, 0.6–1.0). At baseline, frequent stimulant use (1.8, 1.0–3.2) and low educational attainment (0.1, 0.0–0.8) were associated with higher ‘ASRS–memory’ scores. In the subsample fulfilling the ASRS–screener, 45% of the patients were ‘ASRS–positive,’ of whom 13% with a registered ADHD diagnosis.

**Conclusions:**

Our findings illustrate a relationship between the ASRS–memory and –attention scores and frequent cannabis and stimulant use. Furthermore, nearly half of the subsample was ‘ASRS–positive.’ Patients receiving OAT might benefit from being further assessed for ADHD, but improved diagnostic methods are required.

**Supplementary Information:**

The online version contains supplementary material available at 10.1186/s12888-023-04980-w.

## Background

Attention-deficit/hyperactivity disorder (ADHD) is a life-spanning neurodevelopmental disorder associated with three core symptom clusters: inattention, hyperactivity, and impulsivity (ADHD symptoms) [1]. Among patients with substance use disorder (SUD), co-existing ADHD or ADHD symptoms influence outcomes and treatment needs [2]. Compared to patients with SUD without ADHD, those with SUD and ADHD have a higher risk of earlier onset of substance use, drop-out of SUD treatment, and polysubstance use [3–6]. In addition, co-existing ADHD and SUD is associated with higher rates of hospitalizations, suicide attempts, childhood trauma, self-medication with central stimulants, homelessness, low educational attainment, and comorbid mental disorders, including antisocial and borderline personality disorders, and mood disorders [7–14]. Furthermore, the ADHD symptoms are unspecific and may reflect a wide range of medical- and psychosocial conditions other than ADHD. Understanding the link between ADHD symptoms, with or without the diagnosis of ADHD, and associated clinical and sociodemographic variables in patients with SUD may optimize patient management.

In European countries, the estimated prevalence of ADHD varies significantly among patients seeking substance treatment, with the lowest estimated prevalence in Eastern and Southern countries (e.g., 5% in Hungary and 9% in Spain) and the highest estimated prevalence in Northern countries (e.g., 21% in Norway, 20% in Sweden) [2]. Patients with alcohol and opioid dependence are more likely to have ADHD than those with cocaine dependence [15, 16]. Moreover, ADHD symptoms affect up to 33% of patients with SUD [17–19], which substantially exceeds the estimated prevalence of ADHD in this population. Even though a diagnosis of ADHD may explain these symptoms, difficulties in memory and attention in particular, they may also be due to other complex and compound challenges, like psychosocial conditions (e.g., housing situation, imprisoning, financial worries), substance use, and other mental disorders among patients with SUD [7–14, 20]. However, the potential impact of these challenges on memory and attention among patients with SUD is unclear [21–25].

Opioid agonist therapy (OAT) is a well-documented treatment for opioid dependence, particularly long-term heroin dependence [26]. Up to one-third of SUD patients receiving OAT are estimated to meet the criteria for ADHD. However, the prevalence varies between studies, assumingly because having ADHD symptoms without fulfilling diagnostic criteria for ADHD are common in the SUD population [15, 27–30]. Among Norwegian patients receiving OAT, a third were found to have ADHD symptoms needing further diagnostic assessment [17]. In 2017, 4% of Norwegian patients receiving OAT and 18% of Swedish patients receiving OAT were prescribed central stimulants for ADHD [31, 32]. This indicates that, compared to the estimated ADHD prevalence, there are substantial differences in the prescription practice of pharmacological agents for ADHD among patients receiving OAT in Northern European countries. Furthermore, as reported in a Norwegian study [24], among patients receiving OAT who were treated for ADHD, nearly 50% of patients discontinued ADHD medications within 2 years after initiating treatment. The reasons cited for discontinuation were illegal substance use, restrictions on driving licenses, side effects, and medications not meeting patients’ expectations [33]. To date, most ADHD and SUD studies have focused on patients with SUD in general, not specifically those receiving OAT [19, 30, 34]. Thus, there is an urgent need to identify the prevalence of ADHD symptoms, their relationship to a diagnosis of ADHD, and how other clinical and sociodemographic challenges are associated with difficulties in memory and attention among SUD patients receiving OAT.

While diagnostic assessment of ADHD may be difficult to complete in the OAT facilities, a survey of ADHD symptoms is assumingly more feasible. The adult ADHD self-report scale version 1.1 (ASRS) is a validated screening instrument for ADHD [35], with acceptable sensitivity and moderate specificity on SUD populations [36]. The ASRS is subdivided into parts A and B. Part A serves as a screener for ADHD, and part B offers more detailed information valuable for further diagnostic assessment. The prevalence of positive screening on the ASRS, part A, varies from 8 to 37% in different populations [17–19, 36]. The ASRS may be suitable to identify ADHD symptoms and their changes over time in the OAT populations.

The objectives of this study were to investigate memory and attention symptoms and their associations with frequent substance use and sociodemographic characteristics among patients with SUD receiving OAT. Further, in a subsample, we estimated the prevalence of positive screening on the ASRS, part A, and its correlation to having a registered clinical diagnosis of ADHD.

## Methods

### Data sources

We used data from nested cohorts from the INTRO-HCV and ATLAS4LAR studies in Bergen, Norway [37], approved by Helse West and regional ethical committee, see details in the Declaration section (Sect. “Declarations”). Data were collected from May 2017 to March 2022. All the patients were recruited from OAT outpatient clinics. The patients were receiving OAT, meaning they 1) met the criteria for opioid dependence syndrome according to the International Classification of Diseases and Related Health Problems, version 10 (ICD-10), and 2) received OAT opioids daily during the study period. All the patients were older than 18 years.

### Data collections

All consenting patients underwent assessment for ADHD symptoms, substance use, and injecting substance use during annual assessment visits. Sociodemographic conditions were also recorded during these annual assessments. The data were collected by trained research nurses and stored in a health register using electronic data collection software, CheckWare (CheckWare AS, Trondheim, Norway). Clinical data––including information on educational attainment, substance use, injecting substance use, housing status, and mental disorders, were collected from the patients’ electronic medical records. In total, 701 patients receiving OAT were recruited and consented to be included in the study period. The mean time that the patients had received OAT was 9 years, with a standard deviation (SD) of 6 years.

### The adult ADHD self-report scale

**Fig. 1:**
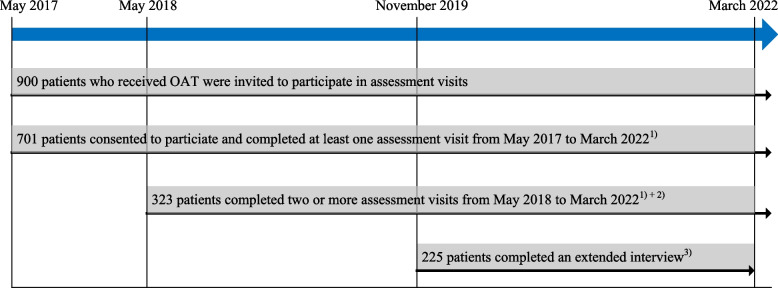
Timeline of data collection of the study sample. OAT: Opioid agonist therapy; ASRS: The adult ADHD self-report scale, version 1.1. ^1)^ Assessment visit: ASRS, part A, question 3, ASRS, part B, question 9, substance use, injecting substance use, sociodemographic factors. ^2)^ A total of 323 of the 701 patients completed the assessment visits twice or thrice during the study period. The remaining 378 patients had completed one assessment visit at the end of March 2022. ^3)^ Of the 701 patients who had completed at least one assessment visit, 225 patients participated in an extended interview during the period from November 2019 to March 2022. Extended interview: ASRS, part A, and information on registered mental disorder diagnoses from medical records

The Adult ADHD Self-Report Scale-v.1.1 is an 18-question validated screening instrument widely used for measuring ADHD symptoms and changes in these symptoms over time. It is based on the diagnostic criteria of the Diagnostic and Statistical Manual of Mental Disorders of the American Psychiatric Association. The ASRS is subdivided into two parts, A and B, with six and 12 questions, respectively, responded to on a Likert scale ranging from never (0) to very often (4). The ASRS, part A, consists of four questions on inattention symptoms (questions 1–4) and two questions on hyperactivity-impulsivity (questions 5–6) [35]. Part A serves as a screener for adult ADHD (ASRS–screener), with sensitivity and specificity varying somewhat between the studied populations [35, 36, 38]. In the present study, during annual assessment visits, 900 patients were asked to respond to at least two questions about memory and attention from the ASRS, parts A and B (Fig. 1). These questions measure two ADHD symptoms that often are affected among patients using substances [21, 24, 25]. The two questions were as follows:

1. How often do you have problems remembering appointments or obligations? (part A, question 3)
2. How often do you have difficulty concentrating on what people say to you, even when they are speaking to you directly? (part B, question 9)

Of the 900 patients questioned, 701 patients (78%) responded to ASRS, part A, question 3 (‘ASRS–memory’), and 666 patients (74%) responded to ASRS, part B, question 9 (‘ASRS–attention’). A total of 323 of 701 patients responded to the ‘ASRS–memory’ question on two or more occasions, rendering 440 repeated responses during the study period, with a mean of 1.3 years (SD: 0.8) between the response times for each patient. Similarly, 241 of 666 patients responded to the ‘ASRS–attention’ question on two or more occasions, rendering 257 repeated responses, with a mean of 1.3 years (SD: 0.5) between the response time for each patient. The symptom cutoffs of the ‘ASRS–memory’ and ‘ASRS–attention’ were defined by a tick in a box in the questionnaire (see Additional file 1). From November 2019 to March 2022, an extended interview, including all the questions on the ASRS–screener, was conducted to improve the survey of ADHD symptoms. In this subsample, information on diagnoses of registered mental disorders, based on standardized interviews, questionnaires, and clinical assessments, and the prescription of central stimulants were collected retrospectively from the patients’ electronic medical records. A total of 225 patients completed the extended interview. An affirmative answer to at least four of six questions in the ASRS–screener, i.e., by a tick in a shaded box, serves as the cutoff for a positive screening for ADHD [35, 39], and is used as a recommendation for further diagnostic assessment. Patients scoring above and below the standard cutoff were defined as ‘ASRS–positive’ and ‘ASRS–negative’, respectively. The ASRS questions were self-administered when the patients cooperated, without being substantially affected by substances or other significant psychosocial conditions. The shadings of answering boxes were omitted in the version of the ASRS questionnaire distributed to the patients in this study.

### Definitions of study variables

Scores above cutoffs on the individual ASRS–memory and –attention questions, and the ASRS–screener (‘ASRS–positive’) were defined categorically according to the standard cutoff on the ASRS, as described above (Sect. “The adult ADHD self-report scale”). “Injecting substance use” was defined as having injected any substance at least once during the 30 days leading up to the assessment visit. Similarly, frequent substance use was categorized as using at least one of the substance classes, including alcohol, stimulants (including amphetamines and cocaine), benzodiazepines, cannabis, and opioids, more than weekly during the past year. Patients who did not use substances or used them less than weekly during the past year were categorized as having ‘no frequent use of substance’. Unstable housing status was defined as living in a homeless shelter or with family or friends at any time during the 30 days leading up to the assessment visit. By contrast, stable housing status was considered as having owned or rented housing situations or being incarcerated during the 30 days leading up to the assessment visit. Mental disorders were categorized into five main classes according to ICD-10 codes: psychotic disorders (F20-F29), bipolar disorder (F31), unipolar depressive disorder (F32-F33), anxiety disorder (F40-F41), personality disorder (F60-F61), and hyperkinetic disorder (F90). Prescription of central stimulants was defined as being prescribed methylphenidate, lisdexamphetamine, or dexamphetamine.

### Statistical analyses

Stata/SE 17.0 (StataCorp, TX, USA) was used for descriptive and regression model analyses, and Microsoft Excel (Microsoft Corporation, Washington, USA) was used to create bar charts displaying the distribution of the self-reported scores of the questions ‘ASRS–memory’ and ‘ASRS–attention’, and the ASRS–screener (for the subsample). IBM SPSS version 26.0 (International Business Machines, Chicago, USA) was used for expectation–maximization calculations. For the analysis of the ‘ASRS–memory’ and ‘ASRS–attention’ questions, baseline was defined as the time of the first responses to these questions in the assessment visit. Unless otherwise stated, the threshold for statistical significance was set to *P* < 0.05 for all analyses.

Any missing values in exposure variables, including substances used, housing status, and injecting substance use, were considered “*missing at random*” when running the expectation–maximization algorithm. Missing values were identified in 11% of the exposure variables, and all were replaced with estimated values. The expectation–maximization algorithm for computing data iteratively performed maximum likelihood estimation in the presence of latent variables [40], which is recommended for optimizing regression models.

Ordinal multilevel mixed-effect logistic regression analyses were performed to investigate whether substance use type, age, sex, educational level, housing status, and injecting substance use (exposure variables) were associated with ‘ASRS–memory’ and ‘ASRS–attention’ scores (ordinal outcome variables), respectively, at baseline and to what extent they were associated with changes in the scores in later assessment visits. The exposure variables were kept constant at the baseline level in predicting the level and changes in the outcome variables. To explore whether exposure variable predicted changes in outcomes, interactions between these variables and time were added to the model. All available responses to the ‘ASRS–memory’ and ‘ASRS–attention’ questions were included. Time was defined as years from baseline. In addition, a sensitivity analysis with a combined sum score variable of the responses to the ASRS–memory and –attention questions was performed to consider potentially overlapping symptoms between the questions. Patients who completed one health assessment visit and only responded to one of the two ASRS questions were excluded in this analysis (*n* = 35).

## Results

### Characteristics of the study sample (*n* = 701) at baseline

A total of 502 (71%) patients were males, and the mean age was 44 years (SD: 10) (Table 1). Thirty-two patients (5%) had not completed primary school, and 319 (46%) had primary school listed as their highest educational attainment. During the past 30 days leading up to the first assessment visit, 394 (62%) patients had used cannabis, 339 (53%) had used benzodiazepines, 339 (53%) had used alcohol, 225 (35%) had used amphetamines, 144 (23%) had used opioids, and 33 (5%) had used cocaine. During the past year, 210 (35%) had injected substances.

**Table 1:** Characteristics of patients receiving opioid agonist therapy at baseline

	Patients with extended interview^a^ (*n* = 225)	All patients (*n* = 701)
Age (years), n (%)		
18–30	10 (4)	66 (9)
30–40	54 (24)	199 (28)
40–50	74 (33)	225 (32)
50–60	68 (30)	165 (24)
≥ 60	19 (8)	46 (7)
Mean (SD)	47 (10)	44 (10)
Sex, n (%)		
Male	168 (75)	495 (71)
Female	57 (25)	206 (29)
Educational attainment, n (%)		
Not completed primary school	11 (5)	32 (5)
Primary school (9 years)	100 (45)	319 (46)
High school (12 years)	94 (42)	287 (41)
≤ 3 years of college or university	11 (5)	51 (7)
> 3 years of college or university	6 (3)	12 (2)
Number of years in opioid agonist therapy, mean (SD)	9 (6)	8 (6)
Injected substances the past 30 days, n (%)	51 (28)	210 (35)
Unstable housing status the past 30 days^b^, n (%)	12 (5)	70 (10)
Substance use during the past 30 days^c^, n (%)		
Cannabis	136 (61)	394 (62)
Benzodiazepines	102 (46)	339 (53)
Alcohol	117 (53)	339 (53)
Amphetamines	66 (30)	225 (35)
Opioids (not OAT)	37 (17)	144 (23)
Cocaine	5 (2)	33 (5)
Mean age for the onset of substance use (mean (SD))	13 (3)	13 (3)
Mental disorders, n (%)		
At least one mental disorder^d^	155 (69)	-
Psychotic disorder (F20-F29)	21 (9)	-
Bipolar disorder (F31)	12 (5)	-
Unipolar depressive disorder (F32-F33)	64 (28)	-
Anxiety disorder (F40-F41)	71 (32)	-
Personality disorders (F60-F61)	27 (12)	-
Hyperkinetic disorder (F90)	36 (16)	-
- Prescribed central stimulants^e^	11 (5)	-

*ASRS* Adult ADHD self-report scale, version 1.1; *ICD-10* The international classification of diseases, version 10; *n* Number of patients; *SD* Standard deviation

^a^An extended interview included responses to the whole ASRS, part A (ASRS–screener) and collection of registered mental disorder diagnoses (ICD-10) from the medical records

^b^ ‘Unstable housing status’ was defined as living in a homeless shelter or with family or friends at any time during the 30 days leading up to the health assessment. ‘Stable housing status’ was defined as having owned or rented housing situation or being incarcerated during the 30 days leading up to the health assessment

^c^The number of patients who had used substances at least once during the 30 days leading up to the first assessment visit

^d^A mental disorder was defined according to ICD-10 codes: psychotic disorder (F20-F29), bipolar disorder (F31), unipolar depressive disorder (F32-F33), anxiety disorder (F40-F41), personality disorder (F60-F61), and hyperkinetic disorder (F90)

^e^Central stimulants were defined as being prescribed dexamphetamine, lisdexamphetamine, or methylphenidate

### ASRS–memory and ASRS–attention scores and their associations with sociodemographic factors and frequent substance use

**Fig. 2:**
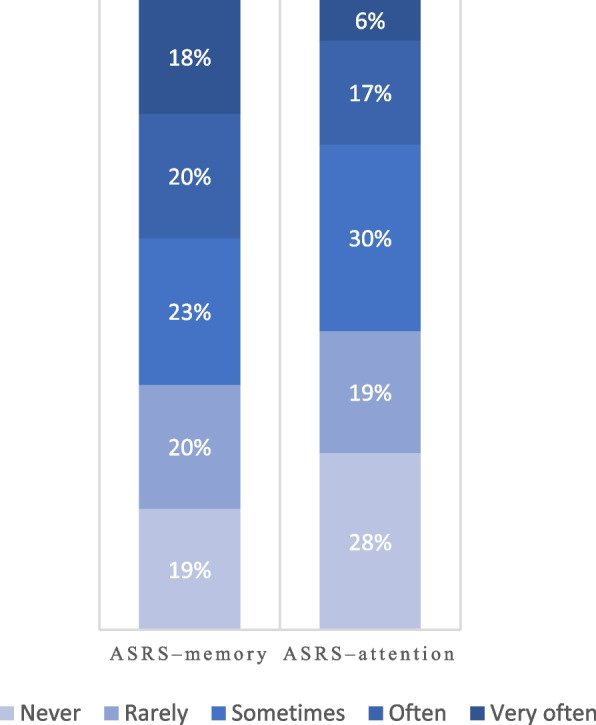
Distribution of responses to the ASRS–memory and –attention at baseline. ASRS: The adult ADHD self-report scale version 1.1. The responses to the ASRS–memory (ASRS, part A, question 3, *n* = 701) and –attention (ASRS, part B, question 9, *n* = 666), presented on a Likert scale ranging from never to very often. ASRS, part A, question 3: *How often do you have problems remembering appointments or obligations?*. ASRS, part B, question 9: *How often do you have difficulty concentrating on what people say to you, even when they are speaking to you directly?* Affirmative answers of “sometimes,” “often,” or “very often” on the scale serves as symptoms over the standard cut-off for these two questions

At baseline, the numbers of patients exceeding the cut-offs for ‘ASRS–memory’ and ‘ASRS–attention’ was 428 (61%) and 307 (53%), respectively (Fig. 2). At baseline, the frequent use of cannabis (odds ratio [OR]: 1.7, 95% confidence interval (CI): 1.1–2.6) and stimulants (amphetamines and cocaine) (OR: 1.8, 95% CI: 1.0–3.2) were associated with a higher ‘ASRS–memory’ score compared with less or no use (Table 2). Likewise, educational attainment was inversely associated with a higher ‘ASRS–memory’ score at baseline (OR: 0.1, 95% CI: 0.0–0.8). However, the frequent use of cannabis compared with less or no use was associated with a tendency towards a slightly reduced ‘ASRS–memory’ score over time (OR: 0.7, 95% CI: 0.6–1.0) and a higher ‘ASRS–attention’ score (OR: 1.7, 95% CI: 1.1–2.5) at baseline. No exposure variables were associated with changes in ‘ASRS–attention’ over time. The sensitivity analysis with a combined ‘ASRS–memory’ and ‘ASRS–attention’ question exposure variable found similar results (Additional file 2), with a correlation between these questions on 0.39.

**Table 2:** Longitudinal ordinal multilevel mixed-effect logistic regression analysis of the association of ASRS–memory and –attention with sociodemographic characteristics and frequent substance use at baseline and over time (per year)

	ASRS-memory^a^ (*n* = 701, number of observations: 1141)				ASRS-attention^b^ (*n* = 666, number of observations: 923)			
	Effect estimate (baseline)		Time trend (per year) (over time)		Effect estimate (baseline)		Time trend (per year) (over time)	
	Odds ratio (95% CI)	*p*-value	Odds ratio (95% CI)	*p*-value	Odds ratio (95% CI)	*p*-value	Odds ratio (95% CI)	*p*-value
Time (per year)	-	-	0.8 (0.4–1.9)	0.666	-	-	1.0 (0.2–4.0)	0.951
*Sex*								
Female	1.0 (0.7–1.6)	0.842	1.1 (0.8–1.5)	0.515	1.3 (0.8–2.0)	0.225	1.0 (0.6–1.7)	0.856
*Age groups*								
18- < 30	1.0 (ref.)	-	1.0 (ref.)	-	1.0 (ref.)	-	1.0 (ref.)	-
30- < 40	0.8 (0.4–1.7)	0.621	1.1 (0.6–2.0)	0.729	0.8 (0.4–1.7)	0.528	0.8 (0.3–2.0)	0.691
40- < 50	1.0 (0.5–2.1)	0.994	1.0 (0.5–1.7)	0.905	0.9 (0.4–2.0)	0.865	0.8 (0.3–1.8)	0.537
50- < 60	0.8 (0.4–1.7)	0.594	0.9 (0.5–1.6)	0.692	0.5 (0.2–1.0)	0.062	0.9 (0.4–2.3)	0.863
≥ 60	0.7 (0.2–1.9)	0.438	1.2 (0.6–2.5)	0.637	0.6 (0.2–1.6)	0.260	1.1 (0.3–3.8)	0.826
*Educational attainment*								
Not completed primary school	1.0 (ref.)	-	1.0 (ref.)	-	1.0 (ref.)	-	1.0 (ref.)	-
Primary school (nine years)	0.4 (0.1–1.0)	0.044	1.5 (0.8–2.9)	0.181	0.9 (0.3–2.4)	0.833	1.5 (0.5–4.6)	0.480
High school (12 years)	0.3 (0.1–0.8)	0.018	1.3 (0.7–2.5)	0.429	0.6 (0.2–1.6)	0.283	1.4 (0.4–4.4)	0.569
≤ 3 years of college or university	0.2 (0.1–0.7)	0.014	0.9 (0.4–2.0)	0.780	0.3 (0.1–1.1)	0.071	0.4 (0.1–2.1)	0.266
> 3 years of college or university	0.1 (0.0–0.8)	0.028	1.0 (0.4–2.4)	0.916	0.3 (0.1–1.8)	0.182	3.0 (0.6–16.2)	0.197
Unstable housing status	1.3 (0.6–2.7)	0.444	1.3 (0.7–2.6)	0.362	1.7 (0.9–3.5)	0.833	2.4 (0.9–6.3)	0.087
Injecting substance use	1.3 (0.8–2.2)	0.251	1.1 (0.8–1.4)	0.752	1.3 (0.8–2.2)	0.241	0.6 (0.4–1.0)	0.071
*Frequent substance use past year*								
Alcohol	1.0 (0.6–1.6)	0.968	1.0 (0.7–1.4)	0.994	1.3 (0.8–2.0)	0.316	1.0 (0.6–1.6)	0.890
Benzodiazepines	1.2 (0.8–1.9)	0.406	0.8 (0.6–1.1)	0.235	1.0 (0.6–1.5)	0.870	1.1 (0.7–1.7)	0.666
Cannabis	1.7 (1.1–2.6)	0.012	0.7 (0.6–1.0)	0.034	1.7 (1.1–2.5)	0.018	0.9 (0.6–1.4)	0.614
Opioids	1.7 (0.9–3.3)	0.100	0.9 (0.5–1.5)	0.711	1.7 (0.9–3.1)	0.128	1.1 (0.5–2.4)	0.755
Stimulants (amphetamines and cocaine)	1.8 (1.0–3.2)	0.043	0.7 (0.5–1.0)	0.053	1.3 (0.8–2.3)	0.315	1.0 (0.5–1.7)	0.912

*ASRS* Adult ADHD self-report scale version 1.1

^a^ASRS, part A, question 3 (ASRS–memory): *How often do you have problems remembering appointments or obligations?* The responses were answered on a Likert scale ranging from never (0) to very often (4)

^b^ASRS, part B, question 9 (ASRS–attention): *How often do you have difficulty concentrating on what people say to you, even when they are speaking to you directly?* The responses were answered on a Likert scale ranging from never (0) to very often (4)

### Characteristics of patients with an extended interview (*n*= 225)

Among the 225 patients who completed an extended interview with the whole ASRS–screener, 168 (75%) patients were males, and the mean age was 44 years (SD: 10). Of these patients, 187 (83%) had used at least one substance during the past 30 days, and 155 (69%) had at least one mental disorder, of which anxiety disorder was present in 71 (32%) patients. A total of 36 (16%) patients were diagnosed with ADHD (F90), of whom 11 (5%) were prescribed central stimulants.

### The relationship between a positive ASRS–screener and registered ADHD diagnosis among patients with an extended interview (*n* = 225)

**Fig. 3:**
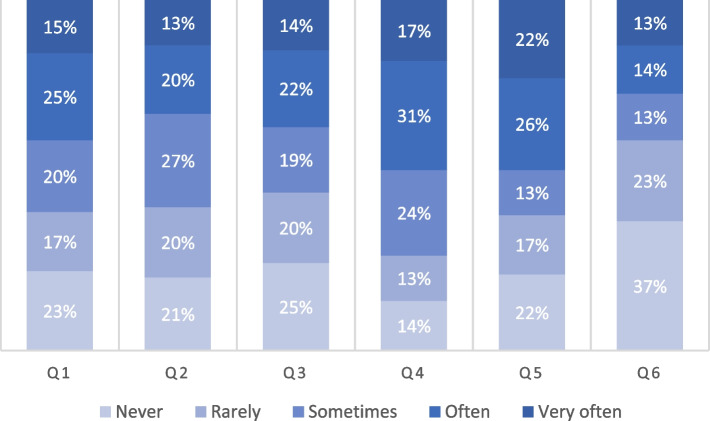
Distribution of responses to the ASRS, part A (*n* = 225). Q: Question; ASRS: The adult ADHD self-report scale version 1.1. The responses to the ASRS, part A, presented on a Likert scale ranging from never to very often. Q1: “*Trouble wrapping up final details*”; Q2: “*Difficulties with organization*”; Q3: “*Problems remembering appointments*”; Q4: “*Delays in getting started task*”; Q5: “*Squirm with your hands or feet*”; Q6: “C*ompelled to do things*”

In total, 101 (45%) patients were ‘ASRS–positive’ according to the standard cutoff for the ASRS–screener, indicating the need for further ADHD assessment (Additional file 3). A total of 36 (16%) of 225 patients had registered a clinical diagnosis of ADHD, according to their medical records. Among these 36 patients, 11 (5%) patients were ‘ASRS–positive’. The numbers of patients who exceeded the standard cutoffs for the individual questions (1–6) were: 136 (60%), 133 (59%), 123 (55%), 109 (48%), 109 (48%), and 61 (27%), respectively (Fig. 3).

## Discussion

More than half of the patients receiving OAT scored over the standard cutoffs on the ‘ASRS–memory’ and ‘ASRS–attention.’ The symptoms were relatively stable over time. Frequent use of stimulants (amphetamines or cocaine) compared to less or no use and having low educational attainment compared to high educational attainment was associated with a higher ‘ASRS–memory’ score. Frequent use of cannabis was associated with higher ‘ASRS–memory’ and ‘ASRS–attention’ scores compared with less or no use, though a slightly reduced ‘ASRS–memory’ score over time. The latter finding could be related to random variation or residual confounding. According to a research report assessing short- and long-term health consequences of methamphetamine use in the United States [21], illegal use of methamphetamines, especially methamphetamines taken in large doses, impairs memory and attention, even long after stopping taking these substances. In our study sample, where nearly a third used amphetamines at least weekly during the past year, the acute exposure and its accompanying impact on memory and attention could be a reason for our findings. Likewise, acute exposure to cannabis, which is known to impair attention and working memory [24, 25], could explain the association between ‘ASRS–memory’ and ‘ASRS–attention’ scores and frequent use of cannabis detected in our study. However, the long-term impact of daily cannabis use on memory is unclear, with studies showing equivocal results and being affected by methodological bias [22, 23, 41]. Thus, our findings on the effects of frequent cannabis use on ASRS–memory, particularly our findings of the reduced score on ASRS–memory over time, must be interpreted with caution.

The prevalence of ‘ASRS–positive’ patients of 45% in the screened subsample in this study was higher than that found in a previous study on patients receiving OAT in Norway (33%) [17] and considerably higher than that reported in studies conducted in Italy (19%) [19] and Taiwan (8%) [18]. However, the latter study used a higher cutoff for being ‘ASRS–positive’. An Australian study of current heroin users found a prevalence of ‘ASRS–positive’ of 31% [42]. The variation in reported prevalence for ADHD symptoms may be because they are non-specific. A comprehensive diagnostic assessment of the ‘ASRS–positive’ patients is necessary to determine whether underlying substance dependencies or other mental disorders best explain the symptoms. Notably, symptoms of substance intoxications or withdrawal and anxiety and mood disorders, in particular, may overlap with symptoms of ADHD [43, 44]. In these cases, even though the symptoms should subside with time or appropriate treatment, poor medical and psychosocial conditions (e.g., low adherence to treatment of mental disorders) and ongoing substance use may maintain these symptoms and affect the diagnostic assessment. The high overall comorbidity among SUD patients may be an essential reason for the high levels of ADHD symptoms detected in this study.

In the screened subsample, 29 (13%) patients who were registered with a clinical diagnosis of ADHD were ‘ASRS–positive,’ and 117 (52%) patients who were not registered with a clinical diagnosis of ADHD were ‘ASRS–negative.’ These findings point to the ASRS having almost similar sensitivity (81%) and specificity (62%) than found previously in studies among patients seeking SUD treatment (sensitivity: 88%, specificity: 67%) but lower specificity than in the general Norwegian population (sensitivity: 80%, specificity: 88%) [36, 38]. In the former study, a sensitivity and specificity of just 74% and 54%, respectively, were calculated among a subsample of patients seeking substance treatment with opioids as their primary substance used. In a general and representative American adult population, the sensitivity and specificity were calculated to be 69% and 99%, respectively, with a total classification accuracy of 98% [35]. In this American population study, patients were initially clinically assessed for ADHD, which could explain the high specificity. However, as a limitation of our finding, the information about a diagnosis of ADHD was collected retrospectively from medical records based on local clinical practice without standardized ADHD assessment. In addition, medical- and psychosocial conditions, referral practice, ongoing substance use, and failure of ADHD assessment could contribute to the prevalence of ADHD being lower than estimated in SUD populations [2]. Even though validation studies of the applicability of the ASRS to patients with opioid dependence, irrespective of whether they are receiving OAT or not, are lacking [36], it is reasonable to assume that the ASRS–screener, by its high sensitivity shown in this population, may be suitable for identifying patients who should be prioritized for further diagnostic assessment for ADHD, even among patients in OAT with comorbid disorders and ongoing substance use. Compared with the estimated prevalence of ADHD among patients with SUD in Northern European countries [2], the proportion of patients with diagnosed ADHD is lower in this study sample. Thus, improved screening, diagnostic assessment, and treatment of ADHD and other mental disorders among patients with SUD are highly required. The ASRS–screener may be a tool in facilitating this, but further studies focusing on how to further assess ADHD in patients with ongoing intake of substances are warranted.

Among the 225 patients who completed the whole ASRS–screener (part A), nearly 70% had at least one registered mental disorder diagnosis, of which unipolar depressive disorder and anxiety disorder dominated. The prevalence of comorbid mental disorders was similar to or even higher in our OAT sample than that of other studies among patients with SUD, where the prevalence of at least one mental disorder varied from 42 to 87% [9, 45, 46]. This might contribute to the high prevalence of ADHD symptoms detected in our OAT sample. However, differentiating ADHD symptoms caused by ADHD from such symptoms caused by other comorbid mental disorders, substance use, and psychosocial conditions (i.e., cognitive impairment and poor nutrition) is clinically challenging. Additionally, information on ADHD symptoms in childhood and adolescence provided by parents and caregivers, which is recommended to confirm a diagnosis of ADHD, is often insufficient and affected by recall bias. Thus, to improve the assessment of ADHD, studies on the association between symptoms of ADHD in childhood and the development of ADHD in adulthood in patients with opioid dependence are needed.

In our study, low educational attainment (not completed primary school) was associated with a higher ‘ASRS–memory’ score compared to high educational attainment (> 3 years of college or university) among patients receiving OAT. This finding supports that of other studies that evaluated ADHD symptoms and academic and educational performance [47]. Relative to the general Norwegian population [48], the educational attainment of the patients in our study receiving OAT was low. The high prevalence of ADHD symptoms in this patient population could explain this finding. ADHD is significantly associated with low educational attainment, presumably due to deficits in inhibition and working memory, motor flexibility, and selective and sustained attention [49]. However, memory difficulties could be confounded by other mental and psychosocial comorbidities in this population not identified by the present study. Thus, due to ASRS being self-reports, ASRS could preferably be performed in cooperation with ASRS-trained clinicians to ensure understanding of ASRS–screener questions and, to some extent, prevent recall bias.

## Strengths and limitations

A major strength of this study was its relatively large sample size of 701 patients receiving OAT, who are typically difficult to reach in healthcare settings. However, one important limitation was that only a subsample of 225 patients completed the whole ASRS–screener, with the remaining patients completing only few questions related to ADHD symptoms. This limited the ability to assess changes in ADHD symptoms over time other than for those about memory and attention. Another limitation of this study was that the ASRS is a self-report questionnaire. Thus, it is unclear to what degree the reported responses on the ASRS–screener, and ‘ASRS–memory’ and ‘ASRS–attention’ questions may be related to other factors, such as substance use, psychosocial conditions and comorbidities. While the ASRS–screener has a sensitivity and specificity of 74% and 54%, respectively, among patients seeking substance treatment with opioids as their primary substance, to our knowledge, no studies have validated the individual ASRS–questions of memory and attention alone, as screening markers for difficulties in these symptom domains. Thus, standardized tests measuring memory and attention may deviate significantly from our findings, which is a limitation in this study. In addition, we lacked information from parents and caregivers on ADHD symptoms during childhood and adolescence in our patient sample, which could also explain the low prevalence of ADHD in this population. Moreover, the diagnoses of mental disorders were collected from the medical records, not based on standardized procedures, which could significantly affect the prevalence of mental disorders in this study. Furthermore, patients’ abilities to remember ADHD symptoms could introduce recall bias. Similarly, remembering and recognizing the consumption of different types of substances and their frequency of use could also be challenging. Thus, the results could be biased for several reasons.

## Conclusion

As documented in this study, more than half of patients receiving OAT scored over the standard cutoffs for ‘ASRS–memory’ and ‘ASRS–attention’. Frequent use of cannabis and stimulants (amphetamines or cocaine) and low educational attainment were associated with a high ‘ASRS–memory’ score. The frequent use of cannabis was associated with a high ‘ASRS–attention’ score. In a subsample, nearly half of the patients fulfilling the whole ASRS–screener exceeded the cutoff indicating the need for further ADHD assessment, of whom 29 patients (13%) had been clinically diagnosed with ADHD at some stage. The ASRS may be suitable to identify ADHD symptoms and thus prioritize patients for further diagnostic assessment, but improved diagnostic methods are required to disentangle overlapping symptoms of ongoing substance use from ADHD.

## Supplementary Information

**Additional file 1. **US-English and Norwegian versions of the Adult ADHD self-report scale version 1.1, part A, and part B, question 9. Legends: ADHD: Attention-deficit/hyperactivity disorder.

**Additional file 2. **Longitudinal ordinal multilevel mixed-effect logistic regression analysis of the association of ASRS–memory and –attention (combined outcome variable) with sociodemographic characteristics and substance use at baseline and over time (per year). Legends: ADHD: Attention-deficit/hyperactivity disorder; ASRS-A: The adults ADHD self-report scale version 1.1, part A. ^1)^ The combined outcome variable was generated as a sum score of the responses to ASRS, part A, question 3 (ASRS–memory) and ASRS, part B, question 9 (ASRS–attention), ranged from 0 to 8. Patients who completed one health assessment visit and only responded to one of the two ASRS questions were excluded (*n* = 35). A total of 666 patients responded to ASRS–memory and attention questions. Of those, 241 had completed two or more occasions, rendering 257 repeated responses to the questions. The correlation between ASRS–memory and –attention questions was 0.39. ASRS–memory: *How often do you have problems remembering appointments or obligations? *ASRS–attention: *How often do you have difficulty concentrating on what people say to you, even when they are speaking to you directly?* The responses were answered on a Likert scale ranging from never (0) to very often (4) for each question, which generated a sum score from 0 to 8.

**Additional file 3. **The relationship between adults ADHD self-report scale version 1.1, part A (ASRS-A), and clinically diagnosed ADHD (presented by numbers in each category). Legends: ADHD: Attention-deficit/hyperactivity disorder; ASRS-A: The adults ADHD self-report scale version 1.1, part A. ASRS-A + and ASRS-A – were defined as exceeding and not reaching the symptom cutoff for at least four of six questions in the ASRS, part A, respectively. ADHD + and ADHD – were defined as being registered or not registered with a diagnosis of ADHD according to medical records. ^1)^ A total of 10 patients were prescribed central stimulants. ^2)^ A total of one patient was prescribed central stimulants. ASRS-A + and ADHD +: Spearman’s rho = 0.3130. Sensitivity = 29/36 = 0.806; Specificity = 117/189 = 0.619.

## Data Availability

The datasets analyzed during the current study are not publicly available due data protection requirements, but anonymous data files could be provided on reasonable request from the corresponding author.

## Abbreviations

ADHD: Attention-deficit/hyperactivity disorder
ASRS: Adult ADHD self-report scale version 1.1
DSM-IV: Diagnostic and Statistical Manual of Mental Disorders IV
ICD-10: The international classification of diseases and related health problems, version 10
OAT: Opioid agonist therapy
SUD: Substance use disorder
