# Supplementary material for: Attention-deficit/hyperactivity disorder (ADHD) symptoms and their relation to diagnosed ADHD, sociodemographic characteristics, and substance use among patients receiving opioid agonist therapy: a Norwegian cohort study
Source: BMC Psychiatry. 2023 Jun 29;23:479. doi: 10.1186/s12888-023-04980-w (PMC10308780; doi:10.1186/s12888-023-04980-w)
Supplement: Supplementary file 1 — Additional file 1. US-English and Norwegian versions of the Adult ADHD self-report scale version 1.1, part A, and part B, question 9. Legends: ADHD: Attention-deficit/hyperactivity disorder. [file 12888_2023_4980_MOESM1_ESM.docx]

Additional File 1

| **Part A** | | | | | |
| --- | --- | --- | --- | --- | --- |
|  | Never (0) | Rarely (1) | Sometimes (2) | Often (3) | Very often (4) |
| Q1: How often do you have trouble wrapping up the final details of a project, once the challenging parts have been done? |  |  |  |  |  |
| Q2: How often do you have difficulty getting things in order when you have to do a task that requires organization? |  |  |  |  |  |
| Q3: How often do you have problems remembering appointments or obligations? |  |  |  |  |  |
| Q4: When you have a task that requires a lot of thought, how often do you avoid or delay getting started? |  |  |  |  |  |
| Q5: How often do you fidget or squirm with your hands or feet when you have to sit down for a long time? |  |  |  |  |  |
| Q6: How often do you feel overly active and compelled to do things, like you were driven by a motor? |  |  |  |  |  |
| **Part B (question 9 only)** | | | | | |
| How often do you have difficulty concentrating on what people say to you, even when they are speaking to you directly? |  |  |  |  |  |

| **Del A** | | | | | |
| --- | --- | --- | --- | --- | --- |
|  | Aldri (0) | Sjelden (1) | I blant (2) | Ofte (3) | Svært ofte (4) |
| S1: Hvor ofte har du problemer med å avslutte en oppgave etter at de interessante delene er unnagjort? |  |  |  |  |  |
| S2: Hvor ofte er det vanskelig for deg å få orden på ting når du skal utføre en oppgave som krever organisering? |  |  |  |  |  |
| S3: Hvor ofte har du problemer med å huske avtaler eller forpliktelser? |  |  |  |  |  |
| S4: Når du har en oppgave som krever at du tenker nøye igjennom det du skal gjøre, hvor ofte unngår eller utsetter du å begynne på den? |  |  |  |  |  |
| S5: Hvor ofte sitter du og fikler med noe når du må sitte lenge i ro? |  |  |  |  |  |
| S6: Hvor ofte føler du deg overdrevet aktiv og tvunget til å gjøre noe, som om du var drevet av en indre motor? |  |  |  |  |  |
| **Del B (kun spørsmål 9)** | | | | | |
| S9: Hvor ofte har du vansker med å konsentrere deg om hva folk sier, selv når de snakker direkte til deg? |  |  |  |  |  |
