# Supplementary material for: Attention-deficit/hyperactivity disorder (ADHD) symptoms and their relation to diagnosed ADHD, sociodemographic characteristics, and substance use among patients receiving opioid agonist therapy: a Norwegian cohort study
Source: BMC Psychiatry. 2023 Jun 29;23:479. doi: 10.1186/s12888-023-04980-w (PMC10308780; doi:10.1186/s12888-023-04980-w)
Supplement: Supplementary file 2 — Additional file 2. Longitudinal ordinal multilevel mixed-effect logistic regression analysis of the association of ASRS–memory and –attention (combined outcome variable) with sociodemographic characteristics and substance use at baseline and over time (per year). Legends: ADHD: Attention-deficit/hyperactivity disorder; ASRS-A: The adults ADHD self-report scale version 1.1, part A. 1) The combined outcome variable was generated as a sum score of the responses to ASRS, part A, question 3 (ASRS–memory) and ASRS, part B, question 9 (ASRS–attention), ranged from 0 to 8. Patients who completed one health assessment visit and only responded to one of the two ASRS questions were excluded (n = 35). A total of 666 patients responded to ASRS–memory and attention questions. Of those, 241 had completed two or more occasions, rendering 257 repeated responses to the questions. The correlation between ASRS–memory and –attention questions was 0.39. ASRS–memory: How often do you have problems remembering appointments or obligations? ASRS–attention: How often do you have difficulty concentrating on what people say to you, even when they are speaking to you directly? The responses were answered on a Likert scale ranging from never (0) to very often (4) for each question, which generated a sum score from 0 to 8. [file 12888_2023_4980_MOESM2_ESM.docx]

Additional File 2

|  | | ASRS–memory/attention^1)^  (n = 666, number of observations: 923) | | | |
| --- | --- | --- | --- | --- | --- |
|  | | Effect estimate (baseline) | | Time trend (per year)  (over time) | |
|  | | Odds ratio  (95 % CI) | *p*-value | Odds ratio  (95 % CI) | *p*-value |
| Time (per year) | - | | - | 1.2 (0.3-4.6) | 0.775 |
| *Sex*  Female | 1.2 (0.8–1.8) | | 0.453 | 1.1 (0.7–1.8) | 0.745 |
| *Age groups*  18-<30  30-<40  40-<50  50-<60  ≥ 60 | 1.0 (ref.)  0.7 (0.3–1.5)  1.0 (0.5–2.1)  0.6 (0.3–1.3)  0.6 (0.2–1.7) | | -  0.390  0.956  0.165  0.319 | 1.0 (ref.)  1.0 (0.4–2.3)  0.8 (0.3–1.9)  1.0 (0.4–2.5)  1.4 (0.5–4.4) | -  0.985  0.636  0.963  0.541 |
| *Educational attainment*  Not completed primary school  Primary school (nine years)  High school (12 years)  ≤ 3 years of college or university  > 3 years of college or university | 1.0 (ref.)  0.5 (0.2–1.4)  0.4 (0.1–1.1)  0.3 (0.1–0.9)  0.2 (0.0-1.0) | | -  0.211  0.065  0.028  0.045 | 1.0 (ref.)  1.1 (0.4–3.3)  0.9 (0.3–2.6)  0.4 (0.1–1.6)  1.1 (0.2–5.5) | -  0.821  0.798  0.178  0.905 |
| Unstable housing status | 1.4 (0.7–2.8) | | 0.370 | 2.4 (0.9–6.5) | 0.071 |
| Injecting substance use | 1.5 (0.9–2.4) | | 0.120 | 0.8 (0.5–1.3) | 0.353 |
| *Frequent substance use past year*  Alcohol | 1.1 (0.7–1.9) | | 0.572 | 0.8 (0.5–1.4) | 0.488 |
| Benzodiazepines | 1.1 (0.7–1.7) | | 0.753 | 1.2 (0.8–1.8) | 0.475 |
| Cannabis | 1.9 (1.2–2.9) | | 0.003 | 0.8 (0.5–1.1) | 0.183 |
| Opioids | 2.0 (1.1–3.9) | | 0.032 | 0.9 (0.4–1.8) | 0.778 |
| Stimulants (amphetamines and cocaine) | 1.7 (1.0–3.0) | | 0.061 | 0.7 (0.4–1.3) | 0.274 |
