# Supplementary material for: Attention-deficit/hyperactivity disorder (ADHD) symptoms and their relation to diagnosed ADHD, sociodemographic characteristics, and substance use among patients receiving opioid agonist therapy: a Norwegian cohort study
Source: BMC Psychiatry. 2023 Jun 29;23:479. doi: 10.1186/s12888-023-04980-w (PMC10308780; doi:10.1186/s12888-023-04980-w)
Supplement: Supplementary file 3 — Additional file 3. The relationship between adults ADHD self-report scale version 1.1, part A (ASRS-A), and clinically diagnosed ADHD (presented by numbers in each category). Legends: ADHD: Attention-deficit/hyperactivity disorder; ASRS-A: The adults ADHD self-report scale version 1.1, part A. ASRS-A + and ASRS-A – were defined as exceeding and not reaching the symptom cutoff for at least four of six questions in the ASRS, part A, respectively. ADHD + and ADHD – were defined as being registered or not registered with a diagnosis of ADHD according to medical records. 1) A total of 10 patients were prescribed central stimulants. 2) A total of one patient was prescribed central stimulants. ASRS-A + and ADHD +: Spearman’s rho = 0.3130. Sensitivity = 29/36 = 0.806; Specificity = 117/189 = 0.619. [file 12888_2023_4980_MOESM3_ESM.docx]

Additional File 3

|  | **ADHD +** | **ADHD –** | **Total** |
| --- | --- | --- | --- |
| **ASRS-A +** | 29^1)^ | 72 | 101 |
| **ASRS-A –** | 7^2)^ | 117 | 124 |
| **Total** | 36 | 189 | 225 |
